# Reported Increase in Substance Use Following Mass Terrorism and the Role of Psychosocial Factors

**DOI:** 10.1001/jamanetworkopen.2024.23993

**Published:** 2024-07-24

**Authors:** Roi Eliashar, Tanya Zilberstein, Dvora Shmulewitz, Vera Skvirsky, Merav Vider, Shaul Lev-Ran

**Affiliations:** 1 Israel Center on Addiction, Netanya, Israel; 2 Department of Communication Studies, Ben Gurion University of the Negev, Beer-Sheva, Israel; 3 Lev Hasharon Medical Center, Netanya, Israel; 4 Sackler Faculty of Medicine, Tel Aviv University, Tel Aviv, Israel

## Abstract

**Importance:**

Research has shown evidence of increased substance use (ISU) in populations affected by mass terrorism; however, less is known regarding how psychosocial factors interact to estimate ISU following such events.

**Objective:**

To examine which factors are associated with reporting ISU, test whether psychological distress (PD) mediated the association between exposure to trauma and reporting ISU, and whether the mediation effect differed among those with prior mental health (MH) difficulties and those without.

**Design, Setting, and Participants:**

A partially representative cross-sectional survey of the adult Jewish population in Israel (aged 18-70 years) was conducted 4 weeks after October 7, 2023 (October 31 to November 5, 2023). A national Web panel using quotas based on national distributions was used. Randomly chosen adult Jewish members were invited to participate until a predetermined sample size was reached.

**Main Outcomes and Measures:**

The study measures included reporting ISU (outcome), PD (mediator), exposure to terror events and sociodemographic characteristics (variables), and prior MH difficulties (moderator). Hypotheses regarding outcome and variables were formulated before data collection.

**Results:**

A total of 7000 invitations were distributed, 2679 individuals consented, 1432 were excluded owing to quota limits, 74 failed attention tests, and 205 opted out, resulting in 968 participants (490 women [50.6%]; mean [SD] age, 41.5 [14.6] years). Hierarchical regression analysis revealed that participants were significantly more likely to report ISU if they had experienced direct exposure (odds ratio [OR], 5.75; 95% CI, 2.53 to 13.05), indirect exposure (OR, 1.84; 95% CI, 1.27 to 2.67), media exposure (OR, 1.22; 95% CI, 1.09 to 1.36), PD (OR, 1.80; 95% CI, 1.44 to 2.25), or previous MH difficulties (OR, 2.76; 95% CI, 1.86 to 4.09). PD partially mediated the association between indirect exposure (b = 0.20; 95% CI, 0.11 to 0.31) and media exposure (b = 0.14; 95% CI, 0.10 to 0.19) and reporting ISU. No evidence was found to support previous MH difficulties as a moderator of these indirect effects (indirect exposure, b = −0.003; 95% CI, −0.28 to 0.28; media exposure, b = 0.01; 95% CI, −0.06 to 0.08).

**Conclusions and Relevance:**

This study contributes to the research on the association of mass terrorism with ISU while shedding important light on the role it may play in the self-medication of PD following exposure, even in those who might have no previous MH difficulties. These insights are crucial for planning essential health services and preventive measures.

## Key Points

**Question:**

Which psychosocial factors are associated with reporting increased substance use (ISU) following mass terrorism, and how do these factors interact?

**Findings:**

In this cross-sectional study of a partially representative sample of 968 Jewish adults conducted 1 month after October 7, 2023, reporting ISU was higher among those exposed to events directly, indirectly, or via the media. Psychological distress partially mediated the association between indirect and media exposure and reporting ISU, whether or not experiencing mental health difficulties before the event was reported.

**Meaning:**

This study contributes to the research on the impact of mass terrorism and the pathways by which it is associated with reporting ISU.

## Teaser

This cross-sectional study examines the psychosocial factors associated with reporting increased substance use following mass terrorism in an adult population in Israel.

## Introduction

On October 7, 2023, Israel experienced an unprecedented terrorist attack, with more than 1200 individuals killed and 240 taken hostage, mostly civilians (including women, children, and elderly individuals), making it one of the deadliest attacks in modern history.^1^ An ongoing war against Hamas followed the attack. Psychological reactions to such events are expected to be widespread and varied,^2^ perhaps amplified by the severity of the events,^3^ its continuing nature,^4^ and coverage by traditional and social media via live broadcasts of the unfolding events. Although most of the research following such events has focused on acute stress reactions, posttraumatic stress disorder (PTSD), and depression,^2,5^ it has also consistently shown evidence for increased substance use (ISU) in populations affected by terrorism during the initial years after the occurrence.^6^

Substance use is believed to serve as a coping mechanism to alleviate or divert attention from psychological distress that arises after experiencing trauma.^7,8^ However, although it may provide temporary relief, it can also impede processes of natural recovery from acute stress and contribute to the risk for prolonged psychological difficulties, including substance use disorders.^9^ Although short-term ISU may be expected after mass trauma,^3^ it is much less clear which factors play a role in such increases.

First, the type of exposure appears to play a role in patterns of substance use after major traumatic events, such as the September 11, 2001, attacks on the World Trade Center (9/11),^6^ natural disasters, epidemic outbreaks (most recently COVID-19), and economic crises.^7,10,11^ ISU has been associated with the level of exposure and proximity to the event.^4,12,13^ Emerging evidence also suggests that exposure to collective trauma through traditional or social media may be related to increased psychological distress and ISU.^14,15,16,17,18^ However, less is known about how exposure affects substance use in a terror attack as widespread and ongoing as this one.

Moreover, not enough is known about the specific processes that affect risk for ISU following such events. This is particularly important because identifying mediating and moderating factors will provide key information about how exposure to trauma might increase risk for substance use,^19^ which will help to design appropriately targeted interventions for prevention, early identification, and treatment. Specifically, psychological distress, which was found to be associated with exposure level years after 9/11 events,^20^ may lead to substance use as a maladaptive coping mechanism, following traumatic events.^7,8^ Thus, psychological distress may mediate the association between trauma exposure and ISU, but, to our knowledge, this hypothesis has not been formally tested in a population sample within a short time after experiencing trauma of unprecedented magnitude and ongoing nature. Furthermore, it has been suggested that preexisting mental health difficulties might play a substantive role in the association between exposure to trauma, psychological distress, and substance misuse and use disorders, with increased risk for substance use in these situations mostly found among those with increased vulnerability.^21^ Therefore, in an adult Jewish population sample, the present study aimed to (1) assess whether substance use increased following the October 7 events; (2) identify which factors were associated with ISU, including demographic factors (eg, age, sex, and family status), event-related factors (direct, indirect and media exposure, evacuation), and psychological factors (current psychological distress or prior mental health difficulties); and (3) test whether psychological distress mediated the association between exposure to trauma and reporting ISU, and whether the mediation effect differed among those with prior mental health difficulties and those without.

## Methods

### Study Design and Participants

The current study followed the American Association for Public Opinion Research (AAPOR) and Strengthening the Reporting of Observational Studies in Epidemiology (STROBE) reporting guidelines. We conducted a partially representative cross-sectional survey of the adult Jewish population (aged 18-70 years) between October 31 and November 5, 2023, 4 weeks after the October 7 events. Data were collected using iPanel, a national digital surveying service with a vast and demographically varied Web panel. Adult panel members who identified their religion as Jewish when signing up for the panel were invited to complete the study questionnaire. Quotas were implemented on the basis of sex, age, religiosity, and geographic district distributions according to data derived from the Israeli Central Bureau of Statistics. Deviations up to 1.0% from quotas were allowed. Data regarding race and ethnicity were not obtained within the current study. The panel randomly invited members eligible to participate according to the quotas until a predetermined sample size was reached. The study was approved by the institutional review board of Reichman University.

A total of 7000 invitations were distributed to panel members, among whom 2679 individuals consented to participate by completing electronic informed consent. Among these, 1432 were excluded because the desired quota had already been reached, 74 failed 1 of the 2 multiple choice attention questions (where participants were required to choose a specific answer), and 205 did not complete the questionnaire.

### Measures

The study used self-reported measures of psychological distress, ISU,^22^ exposure to terror events, and prior mental health difficulties^7^ modified for this study’s specific context. Each of the authors reviewed the questionnaire to ensure its cultural appropriateness and that it captured the current event’s major features.

Self-reported ISU during the month from October 7, compared with the previous month, was assessed using a dichotomous measure, for 5 substances: alcohol, cannabis, sedatives, prescription stimulants, and prescription opioids. ISU was marked as present if the participant responded yes on any of the items.

Exposure to the October 7 events was assessed by 3 separate nonexclusive variables (participants could have more than 1 type of exposure). First, direct exposure was assessed using a dichotomous measure comprising 2 conditions: being present in the places affected by the attack or being exposed to the events as a first responder. Second, indirect exposure was assessed using a dichotomous measure comprising any of 3 conditions: family members or friends of the participant were present in the places affected by the attack, having a personal connection to someone who was harmed or had their loved ones harmed in the events, or working in one of the helping professions (eg, mental health and general health) with victims of the attack.

Third, media exposure was measured by a scale comprising 3 items, each assessing the frequency of encountering a potentially traumatic type of content during the preceding month: uncensored verbal descriptions of events, uncensored images or videos of events, and hate discourse on social media. Each item was scored on a 7-point Likert scale, consisting of the following response options: not at all, once or twice, several times, weekly, several times a week, daily or almost daily, and several times a day. The measure was calculated as the average of the 3 items (mean [SD], 3.84 [1.83]; α = .86).

The Kessler-6^23^ measures nonspecific psychological distress consisting of 6 items. Items assess for symptoms experienced over the past 30 days, including nervousness, hopelessness, and worthlessness. Each item in the scale is scored using a 5-point Likert scale ranging from 0 (none of the time) to 4 (all the time) (mean [SD], 1.73 [0.99]; α = .91).

Previous mental health difficulties were assessed using the question, “Prior to October 7, have you experienced mental health difficulties such as anxiety, depression, post-trauma, etc.?” Demographic characteristics included sex, age, geographic district, religiosity, family status, and occupational status before October 7. The impact of war on familial, social, and occupational status was assessed using a dichotomous item regarding being evacuated from home, an ordered scale regarding changes in work volume, and a dichotomous item regarding the limited access to educational opportunities for children.

### Statistical Analysis

Data analysis was conducted using SPSS statistical software version 29 (IBM). Using the enter method, we used a 2-stage hierarchical logistic regression to investigate the association between the demographic variables and reporting ISU (model 1). In model 2, we also explored the associations between direct and indirect and media exposure to October 7 events, previous mental health difficulties, past month psychological distress, and reporting ISU. Before performing the analysis, we tested the linearity assumption using the Box-Tidwell procedure^24^ and the multicollinearity assumption by correlating the independent variables. The latter was specifically important to verify that the 3 exposure variables represented different exposure types. Both assumptions of the logistic regression analysis were met. Finally, since the data contained no significant outliers (with standardized residuals higher than 2.5), all cases were included in the sample. To further examine the role of distress in the association between the 3 exposure types and reporting ISU, 3 mediation tests were conducted using model 4 in SPSS’s PROCESS macro.^25^ To examine whether mental health conditions before October 7 moderated these mediation effects, moderated mediation tests were conducted using model 7 in SPSS’s PROCESS macro.^25^ Statistical significance was set at *P* < .05 with 95% CIs.

## Results

### Descriptive Data

The sample of this cross-sectional study included 968 participants aged 18 to 70 years (mean [SD], 41.5 [14.6] years), of whom 490 (50.6%) were women, 578 (59.7%) were married, and 440 (45.5%) held an academic degree (Table 1). Of the participants, 34 (3.5%) reported being directly exposed to the October 7 events, 483 (49.9%) reported being indirectly exposed to the October 7 events, and 313 (32.3%) reported being exposed to harsh content or discourse via media several times a week or more. Seventy-six (7.9%) had been evacuated because of the war, 249 (25.7%) had no educational settings for their children, and 198 (20.5%) had experienced a decrease in work volume because of the war. A total of 740 individuals (76.4%) reported using 1 or more substances in their lifetime, 589 (60.8%) reported using 1 or more substances in the month before October 7, and 184 (19.0%) reported ISU for 1 or more substances in the month following the events, with alcohol being the most common substance for which increase was reported, followed by sedatives, cannabis, prescription opioids, and prescription stimulants.

**Table 1.** Sample Characteristics Before and After October 7, 2023

Variable	Participants, No. (%) (N = 968)
Sex	
Male	478 (49.4)
Female	490 (50.6)
Residency (districts)	
North	152 (15.7)
Haifa	94 (9.7)
Center	335 (34.6)
Tel Aviv	108 (11.2)
Jerusalem	100 (10.3)
South	148 (15.3)
Judea and Samaria	31 (3.2)
Evacuated after October 7, 2023	76 (7.9)
Age, y	
18-24	149 (15.4)
25-34	210 (21.7)
35-44	208 (21.5)
45-44	171 (17.7)
55-64	147 (15.2)
65-70	83 (8.6)
Marital status	
Single	241 (24.9)
Married	578 (59.7)
Divorced	70 (7.2)
Widowed	14 (1.4)
In a relationship	65 (6.7)
Has children	648 (66.9)
Children currently not in an educational setting	249 (25.7)
Education	
Elementary school	13 (1.3)
High school without a matriculation certificate	114 (11.8)
High school with a matriculation certificate	202 (20.9)
Certification studies	199 (20.6)
Bachelor’s degree	310 (32.0)
Master’s degree and higher	130 (13.4)
Employment after October 7, 2023	
No	304 (31.4)
Lower work volume	198 (20.5)
Same work volume	401 (41.4)
Greater work volume	65 (6.7)
Lifetime use	
Alcohol	682 (70.5)
Cannabis	220 (22.7)
Sedatives	125 (12.9)
Prescription stimulants	109 (11.3)
Prescription opioids	78 (8.1)
Any substance	740 (76.4)
Use in the month before October 7, 2023	
Alcohol	526 (54.3)
Cannabis	98 (10.1)
Sedatives	70 (7.2)
Prescription stimulants	52 (5.4)
Prescription opioids	49 (5.1)
Any substance	589 (60.8)
Experienced mental health conditions before October 7, 2023	222 (22.9)
Exposure to the October 7, 2023, events	
Direct exposure	35 (3.5)
Indirect exposure	483 (49.9)
Increase in any substance use after October 7, 2023	
Alcohol	113 (11.7)
Cannabis	38 (3.9)
Sedatives	56 (5.8)
Prescription stimulants	12 (1.2)
Prescription opioids	20 (2.1)
Any substance	184 (19.0)

### Factors Associated With Reporting ISU

The hierarchical logistic regression model revealed that participants were significantly more likely to report ISU following the events if they had direct exposure (odds ratio [OR], 5.75, 95% CI, 2.53-13.05; *P* < .001), indirect exposure (OR, 1.84, 95% CI, 1.27-2.67; *P* < .001), or media exposure (OR, 1.22 for each level of increase in frequency; 95% CI, 1.09-1.36; *P* < .001) (Table 2). Both current psychological distress (OR, 1.80; 95% CI, 1.44-2.25; *P* < .001) and previous mental health difficulties (OR, 2.76, 95% CI, 1.86-4.09; *P* < .001) were significantly associated with reporting ISU. Older age and female sex were also significantly associated with reporting ISU, once exposure and mental health measures were accounted for. Finally, people who were evacuated from home because of the war were also significantly more likely to report ISU, but there was no significant association after adjusting for exposure and mental health measures.

**Table 2.** Hierarchical Logistic Regression to Estimate the Increase in Substance Use

Step and variable	B (SE)	Wald	*df*	*P* value	Exp(B)
Step 1					
Age (continuous)	−0.00 (0.01)	0.07	1	.80	1.00
Male sex	−0.10 (0.17)	0.34	1	.56	0.91
Has children	−0.18 (0.22)	0.67	1	.41	0.84
Children currently not in an educational setting	−0.18 (0.24)	0.56	1	.45	0.83
Evacuated after October 7, 2023	0.80 (0.26)	9.12	1	<.001	2.22
No employment after October 7, 2023		8.39	3	.04	
Lower work volume after October 7, 2023	0.36 (0.22)	2.65	1	.10	1.44
Same work volume after October 7, 2023	−0.25 (0.21)	1.49	1	.22	0.78
Greater work volume after October 7, 2023	−0.18 (0.37)	0.24	1	.63	0.84
Constant	−1.21 (0.32)	14.52	1	<.001	0.30
Step 2					
Age (continuous)	0.02 (0.01)	4.88	1	.03	1.02
Male sex	−0.58 (0.16)	8.72	1	<.001	0.56
Has children	−0.16 (0.24)	0.48	1	.49	0.85
Children currently not in an educational setting	−0.29 (0.26)	1.22	1	.27	0.75
Evacuated after October 7, 2023	0.30 (0.31)	0.94	1	.33	1.35
No employment after October 7, 2023		5.35	3	.15	
Lower work volume after October 7, 2023	0.42 (0.24)	3.02	1	.08	1.53
Same work volume after October 7, 2023	−0.10 (0.23)	0.18	1	.67	0.91
Greater work volume after October 7, 2023	0.15 (0.40)	0.14	1	.70	1.16
Direct exposure	1.75 (0.42)	17.49	1	<.001	5.75
Indirect exposure	0.61 (0.19)	10.30	1	.001	1.84
Exposure via the media	0.20 (0.06)	12.75	1	<.001	1.22
Experienced mental health conditions before October 7, 2023	1.01 (0.20)	25.63	1	<.001	2.76
Kessler-6	0.59 (0.11)	26.62	1	<.001	1.80
Constant	−4.59 (0.52)	77.46	1	<.001	0.01

### Psychological Distress as a Mediator

For those directly exposed to October 7 events, no significant evidence was found for current psychological distress to mediate the association between exposure and reporting ISU (indirect effect of mediation model, b = −0.05; 95% CI, −0.33 to 0.23) (Table 3). However, psychological distress was found to mediate the association between indirect exposure and reporting ISU (indirect effect, b = 0.20; 95% CI, 0.11 to 0.31) and exposure via media and reporting ISU (indirect effect, b = 0.14; 95% CI, 0.10 to 0.19).

**Table 3.** Mediation Effects

Outcome	b (95% CI)
Outcomes of Kessler-6 mediation of the association between direct exposure and the increase in substance use	
Path a	−0.06 (−0.40 to 0.28)
Path b	0.78 (0.59 to 0.96)
Direct	1.62 (0.88 to 2.37)
Indirect (mediation)	−0.05 (−0.33 to 0.24)
Outcomes of Kessler-6 mediation of the association between indirect exposure and the increase in substance use	
Path a	0.28 (0.16 to 0.40)
Path b	0.72 (0.54 to 0.90)
Direct	0.53 (0.19 to 0.88)
Indirect (mediation)	0.20 (0.11 to 0.31)
Outcomes of Kessler-6 mediation of the association between exposure via the media and the increase in substance use	
Path a	0.22 (0.19 to 0.26)
Path b	0.61 (0.42 to 0.81)
Direct	0.18 (0.08 to 0.28)
Indirect (mediation)	0.14 (0.10 to 0.19)

### Previous Mental Health Difficulties as a Moderator

No evidence was found to support previous mental health difficulties as a moderator of the indirect effects of indirect exposure (b = −0.003; 95% CI, −0.28 to 0.28) or media exposure (b = 0.01; 95% CI, −0.06 to 0.08); that is, distress following exposure to October 7 events indirectly or via media mediating the association with reporting ISU was not significantly different for people who reported previous mental health difficulties and for those who did not. In other words, there was no evidence that the association of trauma exposure with reporting ISU through higher distress levels was only found among those reporting prior mental health difficulties.

## Discussion

This cross-sectional study sought to describe patterns of reporting ISU within the first month after the October 7 mass terror attack in Israel and to identify and explore factors associated with reporting ISU. Almost one-fifth of the respondents reported ISU for 1 or more addictive substances following the events. The risk of reporting ISU was increased among those exposed to events directly, indirectly, and via the media. Experiencing psychological distress in the weeks since the event was higher among those exposed indirectly and via media and was also associated with reporting ISU, with significant indirect effects, thus partially mediating the association between those types of exposure and reporting ISU. However, there was no significant evidence to support the mediation of the direct exposure effect by distress. Furthermore, although experiencing mental health difficulties before the event was associated with reporting ISU, there was no evidence that mental health difficulties modified the indirect associations between indirect and media exposure and reporting ISU via current psychological distress. After controlling for exposure and mental health measures, older age and female sex were also associated with reporting ISU, as was being evacuated from home because of the war, although this association was attenuated by exposure and mental health factors.

The ISU following mass terrorism and other large-scale traumatic events is consistent with previous research.^3^ For example, a meta-analysis^6^ based on 31 population-based studies, most of them following the 9/11 events, found that in the first 2 years following the event, 7.3% of the population reported increased alcohol use, and 16.3% of the population reported increased mixed drug use. Exposure level was also associated with ISU. This resonates with current findings showing that direct exposure has a larger association with reporting ISU than does indirect exposure. Furthermore, even though the *Diagnostic and Statistical Manual of Mental Disorders* (Fifth Edition) specifically elaborates that exposure through media, whether electronic or through television, movies, or pictures, should not be accounted for the diagnosis of PTSD unless it is done constantly as part of a profession; the association found in this study between media exposure and reporting ISU suggests it might also be an important factor in the transmission of trauma.^26,27^ Other studies^28,29^ on media exposure found that both the volume of exposure and explicitness of the content were related to PTS symptoms while highlighting the role social media might play in trauma exposure owing to its more personalized, biased, and explicit nature. Further studies are needed to determine when and what types of media exposure could be considered a traumatic event in terms of PTSD, especially as social media is becoming increasingly more central in the transmission of information and news, specifically in emergency situations like terrorist attacks.^16^

As observed, current psychological distress was associated with reporting ISU, and distress partially mediated the association between indirect exposure and exposure via media and reporting ISU. This is in line with other studies identifying substance use as a possible self-medicating mechanism to cope with psychological distress following trauma.^8^ Similarly, in a 3-wave longitudinal study done in Israel during years with frequent terrorism events, Massey et al^19^ found that depressive symptoms in wave 2 mediated the association between exposure to terrorism and past stressful life events in wave 1 and the frequency of alcohol use in wave 3. However, they also found that PTSD symptoms following exposure were negatively associated with alcohol use frequency, possibly because of the focus on frequency of consumption rather than quantity. In this sense, our study highlights the importance of looking at channels between trauma exposure and substance use beyond stress symptoms, accounting for an increase in quantity and not just frequency, and exploring other substances besides alcohol.

The absence of evidence indicating that distress mediated the association between direct exposure and reporting ISU could be due to the study sample size and the low prevalence of direct exposure to the events in the sample. Otherwise, stress reactions might differ according to exposure type. Further research is required to comprehend the interplay between psychological symptoms and substance use in the context of different types of exposure to traumatic events such as terrorism.

Furthermore, other studies have highlighted how individuals in the population with mental health issues might be more inclined to use substances as a coping mechanism in stressful times^4^ and how preexisting vulnerabilities play a role in the development of PTSD following trauma and subsequent substance use disorders.^30^ Although this study found that reporting previous mental health difficulties was associated with reporting ISU, no evidence was observed that difficulties modified the indirect effect through current distress. This might imply that the experience of trauma in such severe incidents operates in a distinct manner and has consequences that go beyond preexisting vulnerability. Another explanation might be that the dichotomous measure of reported mental health difficulties might not have fully accounted for participants’ mental health vulnerabilities. Further research is required to understand the progression of psychological distress and heightened substance use following a traumatic event, as well as the implications of vulnerability in these processes.^3^

Surprisingly, older age and female sex were associated with reporting ISU once trauma and mental health factors were accounted for. Although young adults and men are more likely to use substances as a coping mechanism,^6^ life circumstances for young men in wartime might allow less use of substances. Furthermore, women and older persons exhibit a higher prevalence in the use of prescription pharmaceuticals, which could potentially contribute to their responses to mass trauma and catastrophes.^31,32^ Evacuation could potentially increase the chance of reporting ISU, because it involves experiencing trauma and heightened distress. Nevertheless, the evacuation occurred in close proximity to the events, resulting in substantial overlap between the individuals who were evacuated and those who were directly or indirectly exposed to trauma. This increases the likelihood that any observed differences can be attributed to the exposure rather than to the evacuation (although the consequences of the latter might be more prominent over time).

### Limitations

This study was subject to 4 main limitations. First, the study was conducted with a partially representative sample of internet panel users, using self-report questionnaires, with certain matters addressed retrospectively. This may involve biases such as underrepresenting population groups with low digital proficiency, self-selection of participants, insufficient reporting of difficulties, and bias in recalling information. This limitation was partially addressed by the use of a nationally diverse panel, the establishment of quotas, and the verification of attention. However, data on ethnic origin were not collected. These variables should be addressed in future studies. Second, this study used a cross-sectional design, which restricts the ability to control the direction of some associations. This limitation implies that the association between distress, media exposure, and substance use may operate in more intricate manners, such as the possibility of substance use leading to higher distress and media exposure. Longitudinal and prospective studies are necessary for evaluating preexisting conditions and the progression of these processes.

Third, previous mental health difficulties were assessed using the question: “Prior to October 7, have you experienced mental health difficulties such as anxiety, depression, post-trauma, etc.?” Using this formulation, the question did not define whether participants had previously received a formal diagnosis of a mental disorder and also did not define a specific time frame for the difficulties experienced. These might lead to a broad range of interpretations of this question by participants. Hence, future studies should address variations in the mental health definitions, as well as additional variables that may play a role in the increase in substance use after the events, such as prior substance use disorder diagnosis, and interpersonal differences such as impulsivity and external locus of control,^33^ data on which were not collected in this study. Fourth, investigating the specific associations related to each type of substance was beyond the scope of the study. This should be addressed in future studies, because the mechanisms related to the use of different substances following exposure to traumatic events might differ.

## Conclusions

This cross-sectional study contributes to the existing body of research on the impact of mass terrorism and war on the ISU among the Jewish adult population during the initial weeks after the beginning of the events. It added important insights regarding the outcomes of psychological distress on these consequences and emphasized the potential prevalence of distress and ISU among individuals exposed to events of such scope and to unparalleled media attention, independent of their preexisting vulnerability. These insights are crucial for planning and disseminating essential health services and preventative measures.

## Notes

Supplement.
Data Sharing Statement
