# Supplementary material for: Reported Increase in Substance Use Following Mass Terrorism and the Role of Psychosocial Factors
Source: JAMA Netw Open. 2024 Jul 24;7(7):e2423993. doi: 10.1001/jamanetworkopen.2024.23993 (PMC11270133; doi:10.1001/jamanetworkopen.2024.23993)
Supplement: Supplement. — Data Sharing Statement [file jamanetwopen-e2423993-s001.pdf]

Data Sharing Statement

Eliashar. Reported Increase in Substance Use Following Mass Terrorism and the Role of Psychosocial Factors. *JAMA Netw Open*. Published July 19, 2024.
doi:10.1001/jamanetworkopen.2024.23993

Data
Data available: No

Additional Information
Explanation for why data not available: Data may be shared upon request from the author, according to ethical considerations.
